# Late Onset Traumatic Diaphragmatic Herniation Leading to Intestinal Obstruction and Pancreatitis: Two Separate Cases

**DOI:** 10.1155/2015/549013

**Published:** 2015-08-24

**Authors:** Tolga Dinc, Selami Ilgaz Kayilioglu, Faruk Coskun

**Affiliations:** Department of General Surgery, Ankara Numune Training and Research Hospital, Anafartalar Mah, Talatpasa Boulevard No. 5, Genel Cerrahi AD, 2. Kat B216, Altındağ, 06100 Ankara, Turkey

## Abstract

Although diaphragmatic injuries caused by blunt or penetrating trauma are rare entities, they are the most commonly misdiagnosed injuries in trauma patients and occur in approximately 3–7% of all abdominal or thoracic traumas. Acute pancreatitis secondary to late presenting diaphragmatic hernia is very rare. Here we present two separate cases: one with acute bowel obstruction and the other with acute pancreatitis secondary to late onset traumatic diaphragmatic hernia (three and twenty-eight years after chest trauma, resp.).

## 1. Introduction

Although diaphragmatic injuries caused by blunt or penetrating trauma are rare entities, they are the most commonly misdiagnosed injuries in trauma patients and occur in approximately 3–7% of all abdominal or thoracic traumas [1]. When these injuries are not diagnosed immediately, they present months or years after the initial trauma in about 9.5% to 61% of all cases [2]. These late presentations can result in pulmonary complications, chronic abdominal pain, or acute bowel obstruction, which might cause morbidity and mortality [3]. However, acute pancreatitis secondary to traumatic diaphragmatic hernia (TDH) is quite rare.

Here we present two separate cases: one with acute bowel obstruction and the other with acute pancreatitis secondary to late onset TDH (three and twenty-eight years after chest trauma, resp.).

## 2. Case Presentation

*Case 1*. A 33-year-old male patient presented with symptoms of abdominal pain, vomiting, and obstipation to our emergency surgery department.

His vital signs at admission were normal besides the pulse rate of 110/min. His physical examination revealed decreased breathing sounds and a healed scar on the left hemithorax. Patient had a history of penetrating thoracic trauma through the intersection of left anterior axillary line and costal arch, three years ago. The patient rejected any surgical intervention at that time and left the emergency clinic. In his current presentation initial evaluation was quite helpful in identification of the source of ileus. Laboratory levels were within normal ranges besides elevated white cell count of 18.3 (×10^3^/*μ*L). Abdominal plain X-ray showed elevation of left diaphragm and infiltration in the left lung. This leaded to a computed tomography (CT) of chest which demonstrated herniation of small bowel, transverse, and descending colonic segments into left hemithorax through a 4 cm defect on diaphragm.

Patient underwent an urgent laparotomy which revealed a posterolateral diaphragmatic defect of 6 cm in diameter with herniation of loops of small and large intestines into the left thoracic cavity. Intestines were not ischemic; thus no resection was performed. After reduction of the intestines to the abdomen, the diaphragmatic defect was successfully repaired primarily with a single layer of nonabsorbable sutures. Patient's postoperative recovery was uneventful. Drainage tubes of abdomen and thorax were removed on the postoperative day 4. Patient was discharged on the postoperative day 6 after the surgery.

*Case 2*. A forty-two-year-old male patient presented to our emergency surgery department with abdominal pain and respiratory distress. He reported that the pain started two days ago, along with respiratory distress which gradually worsened. The abdominal pain was reflecting to his back. The patient had a history of being run over by an asphalt paving cylinder 28 years earlier which resulted in paraplegia secondary to vertebral injury. His vital signs were within normal limits.

**Figure 1:**
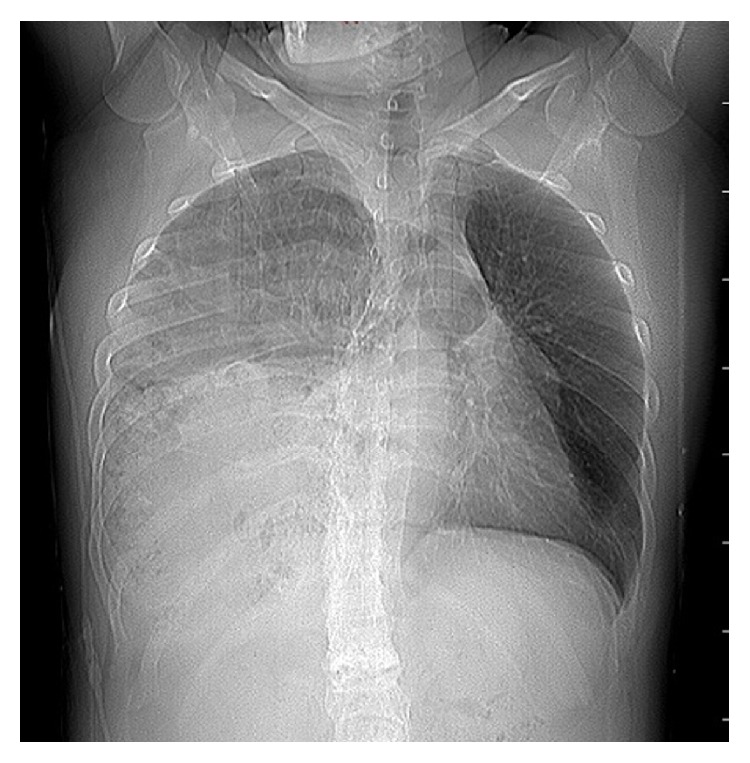
Chest X-ray. Right lung was pushed upwards and right hemithorax was filled with intestines.

**Figure 2:**
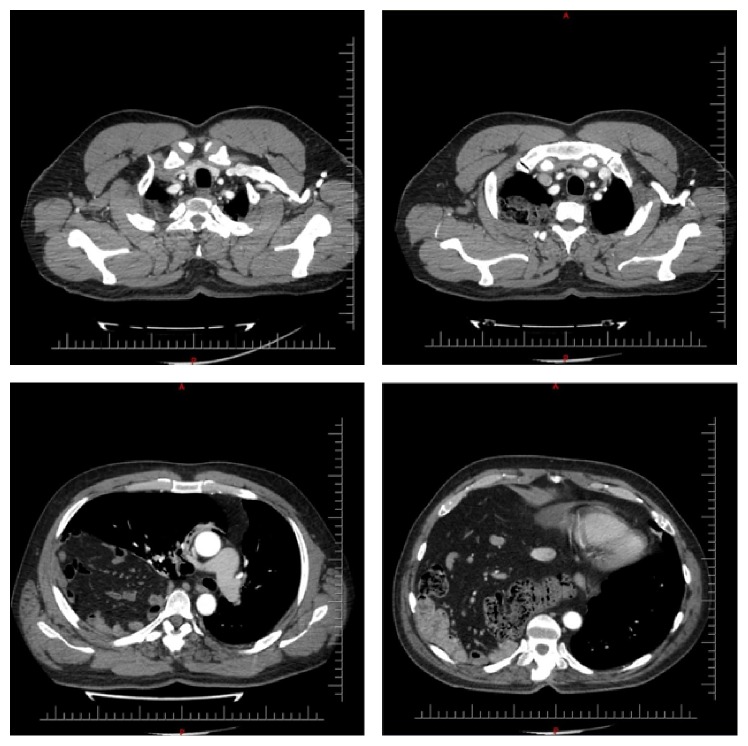
Computerized tomography scan. A defect on the right hemidiaphragm and small bowels, ascending colon, cecum, superior mesenteric artery and branches, pancreas, and duodenum were inside right hemithorax.

In his biochemical analysis amylase level was 2209 U/L, aspartate aminotransferase level was 135 U/L, alkaline phosphatase was 134 U/L, gamma glutamyl transferase was 1238 U/L, and total bilirubin level was 4.6 mg/dL. No gallbladder stones were detected in ultrasonography. In his chest X-ray right lung was pushed upwards and right hemithorax was filled with intestines (Figure 1). In computerized tomography it was noted that there was a 15 cm defect on the right hemidiaphragm and small bowels, ascending colon, caecum, superior mesenteric artery and its branches, pancreas, and duodenum were inside the right hemithorax (Figure 2). On his 3rd day in hospital, amylase levels started to return to normal and bilirubin levels became normal. After full recovery from acute pancreatitis, patient underwent surgical repair of the diaphragmatic defect. Despite the long history, herniated organs were easily pulled back to the abdomen without any significant adhesions and the defect was sutured. His postoperative course was uneventful and the right lung fully expended after removal of the chest tube (Figure 2).

## 3. Discussion

Diaphragmatic tear can occur after blunt or penetrating injury. Small diaphragmatic hernias might be realized months or even years later, when the patients become symptomatic. Physicians and surgeons should keep diaphragmatic hernia in mind in patients with a previous history of trauma with atypical abdominal and respiratory symptoms [4]. Blunt and penetrating traumas account for 68–75% and 25–32% of traumatic diaphragmatic injuries, respectively [5]. One of our patients had a right sided TDH 28 years after blunt trauma and the other had a left sided TDH three years after a penetrating injury.

While it is rare, a delayed TDH is an important cause of bowel obstruction. In such cases, diagnosis must be made immediately, so that an operative intervention can be done to prevent tissue necrosis and other complications related with high mortality. 7% to 66% of diaphragmatic injuries in multitrauma patients remain undiagnosed [6]. When appropriate intervention is not undertaken in the acute setting, chronic diaphragmatic hernia and strangulation may occur in some patients, subsequently. This is the exact sequence that occurred in our first case.

Blunt trauma may cause injury in any part of the diaphragm, but the majority of injuries occur in the posterolateral aspect of the left side of the diaphragm [7]. Our second case who had a past history of blunt trauma contradicts with this fact, as the patient had a large right sided defect.

The cases in medical literature about acute pancreatitis after diaphragmatic herniation of the pancreas are mainly related to hiatal hernias or congenital hernias. Nevertheless, even this is an extremely rare presentation with no more than 7 cases described in literature [8–10]. Acute pancreatitis secondary to TDH is even rarer and we could only find one article dating back to 1952 via PubMed search, using “pancreatitis and traumatic diaphragmatic hernia” keywords [11].

There are three theories about the occurrence of acute pancreatitis in diaphragmatic hernia: (1) abnormal traction on the pancreas, total incarceration of the gland in hernia without pancreatic volvulus [12], (2) migration of the pancreas through a hernia sac and repetitive trauma as it crosses the hernia [13], and (3) ischemia associated with stretching of pancreatic vascular pedicle and intermittent folding of the main pancreatic duct [14]. In our Case 2, the pancreas was found in the right hemithorax. This displacement of the pancreas probably caused repetitive trauma and/or intermittent blockage of blood supply and pancreatic duct flow. In this case no gallbladder stones were detected. So TDH remains as the prime suspect for acute pancreatitis.

Operative intervention is the main management for delayed traumatic diaphragmatic hernias, especially if there is an acute complication like strangulation as in our first case. Although the thoracic approach has been recommended for chronic diaphragmatic hernias due to dense intrathoracic adhesions [15], abdominal or a combined chest-abdominal approach may be necessary in cases of strangulation. Laparoscopic surgery is another option in treatment of TDH; however, it can be challenging in cases with too many intra-abdominal adhesions or complicated hernias [16]. In both of our patients abdominal organs were displaced into thorax so our approach was solely abdominal. We did not encounter any thoracic complications, and reduction of hernias was relatively easy, due to absence of intrathoracic adhesions despite chronic presentation.

These two cases point out that the diagnosis of TDH can be overlooked at the initial trauma evaluation, thus stressing out the need to actively examine the diaphragm on all trauma laparotomies. The clinicians need to be vigilant, to maintain a high index of suspicion for the diaphragmatic injuries, whether they are immediately after the trauma or even decades after the trauma. Our case seems to be the second case in medical literature depicting acute pancreatitis after TDH.
