# Expression of Concern on “Late Onset Traumatic Diaphragmatic Herniation Leading to Intestinal Obstruction and Pancreatitis: Two Separate Cases”

**DOI:** 10.1155/2019/6917081

**Published:** 2019-05-22

**Authors:** Case Reports in Emergency Medicine

*Case Reports in Emergency Medicine* would like to express concern with the article titled “Late Onset Traumatic Diaphragmatic Herniation Leading to Intestinal Obstruction and Pancreatitis: Two Separate Cases” published in Case Reports in Emergency Medicine in August 2015 [1], as the article was published without the approval of Dr. Baris D. Yildiz and his name was missing from the authors' list.

An institutional investigation claimed Dr. Yildiz waived his right to be an author, which he disputed. A court of Intellectual and Industrial Property Rights in Turkey ruled that Dr. Yildiz should be listed as co-author, which was upheld at appeal. However, Dr. Yildiz did not agree to a corrigendum listing him as an author.

Additionally, the editorial office was contacted by someone saying they were the legal representative of one of the patients and they had not consented to publication. However, the representative's identity was not verified and they did not respond to queries.
